# “We never boil our milk, it will cause sore udders and mastitis in our cows”- consumption practices, knowledge and milk safety awareness in Senegal

**DOI:** 10.1186/s12889-020-08877-1

**Published:** 2020-05-20

**Authors:** Bhagyalakshmi Chengat Prakashbabu, Jacqueline M. Cardwell, Laura Craighead, Andrée Prisca Ndjoug Ndour, Damitoti Yempabou, Elhadji Ba, Rianatou Bada-Alambedji, Ayayi Justin Akakpo, Javier Guitian

**Affiliations:** 1 Veterinary Epidemiology, Economics and Public Health Group, Department of Pathobiology and Population Sciences, Royal Veterinary College, Hawkshead Lane, Herts, Hatfield, UK; 2 Ecole Inter Etats des Sciences et Médecine Vétérinaires de Dakar (EISMV), Dakar, Senegal; 3 Institut de recherche pour le développement (IRD), Dakar, Senegal

**Keywords:** Milk-borne diseases, Raw milk, Focus-groups, Senegal, Food safety

## Abstract

**Background:**

Milk is a nutrient-rich food that makes an important contribution to diets in several Low and Middle Income Countries such as Senegal. Milk can also harbour several pathogenic microorganisms. As in other low and middle income countries, the dairy industry in Senegal is growing, with an expansion of farms to meet rapidly growing demand in the cities. However, most of the production still happens in the informal sector, and little is known about consumption of milk and milk products, or knowledge, awareness and practices of actors in informal dairy supply chains.

**Methods:**

We conducted structured focus group discussions with dairy farmers and milk processors in three selected regions (Dakar, Thies and Fatick) in Senegal to investigate the consumption practices, awareness of milk borne hazards, and practices relevant to the risk of milk contamination to gain a deeper understanding of drivers of milk-borne diseases. Data on the consumption of milk and milk products were also collected using a closed questionnaire.

**Results:**

Results indicate that milk is an important part of the diet in the study regionsand raw milk consumption is very common. The most common milk product consumed was fermented milk. Awareness of milk borne hazards was limited. Several farmers and processors reported risky practices, despite being aware of better practices, due to cultural beliefs. In households, children, pregnant women and older people were prioritised when milk and milk products were distributed. Dairy farmers and milk processors were more concerned with the lack of food for animals, low production and seasonality of production than the safety of the milk and milk products.

**Conclusions:**

Lack of awareness of milk borne infections and some traditional practices put milk and milk product consumers in the study area at high risk of milk borne diseases.. Prioritising certain sub population at households (Pregnant women and children) makes then vulnerable to milk-borne hazards. It will be challenging to change the risky practices as they are motivated by cultural beliefs hence the best strategy to promote milk safety will be to encourage the boiling of milk by consumers.

## Background

Milk is an important constituent of diets across the globe because of its high nutritional value. It is a key source of protein and micronutrients in the diets of a huge proportion of adults and even more crucial to children in several low and middle income countries (LMICs) [1]. However, milk can also act as a source of several milk borne hazards ranging from bacteria viruses, protozoa and chemical hazards such as antibiotic residues, pesticides and adulterants [2]. Milk borne hazards pose an important health burden in both high and low-middle income countries, but according to the World Health Organisation (WHO), the populations of LMICs in Africa and Asia are particularly affected. Besides, most of this burden is borne by children under 5 years of age [3]. In addition, a recent study by the World Bank estimated that unsafe food costs LMICs 110 billion USD in terms of lost productivity and medical expenses [4]. Even though the burden is highest in Africa, the continent still lacks adequate facilities and tools required for monitoring and controlling milk borne hazards and this is true especially in some West African countries who are recovering from decades of political instability.

In the regions where the burden of food borne illnesses is high, such conditions are often underreported and receive very little policy attention and investments. It is estimated that in industrialised countries, reported food borne illnesses may represent only 1–10% of the real incidence and in LMICs the proportion could be even lower [5]. The social, health and economic impact of foodborne illness are grossly overlooked leading to a lack of adequate public health programmes [6, 7]. This lack of data, control and surveillance programmes leads to the cycle where policymakers continue to ignore the issue. Additionally, there is a rapid change in dietary habits of the global community, which coupled with population growth and improving economic status in LMICs is leading to a rapid increase in the demand for milk and milk products [8]. As more and more people are consuming milk and milk products, there is an urgent need to ensure they are safe.

Some of the key food borne pathogens that are transmitted through milk and milk products have raw milk consumption as the main route of infection to humans [9–11]. Microbial organisms enter milk through different routes. Some pathogenic bacteria are secreted into milk by healthy animals, some are secreted by clinically and subclinically infected animals, and some enter through contamination after milking. Several sources such as milkers’ hands, environment and equipment used for milking, processing and transport can affect contamination levels in milk. Another important factor affecting milk-borne infection isconsumption practices. Pasteurisation and boiling can kill nearly all pathogenic organisms [2]. However, pathogenic microorganisms are sometimes found in pasteurised milk and milk products due to contamination during storage and preparation [12–14]. Hence, practices of all actors in the milk value chain, from farmers to consumers, influence the occurrence of milk borne pathogens in milk and milk products.

Practices of actors involved in food production are influenced usually by their training, knowledge, and beliefs. In informal systems, most partcipants engaged in food production do not have formal training. There is evidence that in such systems people’s actions are affected mostly by beliefs and experience [15]. Currently, there are limited data available on dairy farmers’ knowledge, attitudes and practices on milk borne hazards in sub-Saharan African countries such as Senegal. In order to control pathogens in different stages of production it is important to instigate behavioural change. Behavioural changes can only be achieved if we understand why people act in a certain way and their beliefs associated with it. For example, a study in Nepal used emotional drivers such as nurture and disgust to change food safety behaviours in mothers of young children and found improvement in hygienic practices [16]. Similarly, case studies on milk safety in Sub-Saharan Africa showed that understanding values and cultural beliefs are crucial in food safety risk management and communication.

Senegal is a West African country with a predominantly rural economy. The total human population was about 15 million in 2018 [17]. Agriculture contributes to 14.8% of the Gross Domestic Product (GDP) and the livestock subsector represents 37.3% of the agriculture GDP. In recent years, there has been a steady increase in livestock population followed by an increase in meat and milk supply. However, productivity is still below the average of other LMICs. Milk and milk products are culturally and nutritionally important in Senegal. In 2012, the demand for milk reached 291 million litres [18]. As the local production is very low, Senegal relies on the import of milk powder and other products. According to FAO, in 2012 Senegal’s dairy imports amounted to 121 million USD (FAOSTAT, 2013). In the Senegalese National Plan (PSE), which aims to improve agriculture, livestock and food security (milk, meat, and eggs), the government envisages expansion and modernisation of the dairy sector. These plans are likely to accelerate the already rapid change of the Senegalese dairy sector with the expansion of dairy herds, parallel changes in the breed composition of herds and the ecology of endemic, including milk-borne pathogens of cattle. Hence understanding the drivers for milk-borne diseases is crucial so that disease control strategies can be targeted at critical stages. The aim of the study was to provide an in-depth understanding of the consumption of milk and milk products, knowledge, awareness and practices regarding milk borne infections of dairy farmers and milk processors in Senegal.

In order to address this knowledge gap, we used a combination of qualitative (focus group discussions) and quantitative (closed ended questionnaire) methods in this study. While quantitative tools are useful to ascertain the frequency of behaviours, qualitative methods allow us to explore and understand the motivations, beliefs and principles of behaviours, which are crucial if disease control involves significant behavioural change. Focus group discussions can be used as a powerful tool to elicit information, which can form the basis of future control strategies that are tailored for the beneficiary communities and therefore more likely to be accepted and succeed. The information thus gathered will help to understand risky behaviours if any and facilitate future control programmes. It will enable the policymakers to identify effective strategies to bring about any behavioural changes of key actors involved in the production of milk to ensure safe food for consumers.

## Methods

### Study area

**Fig. 1:**
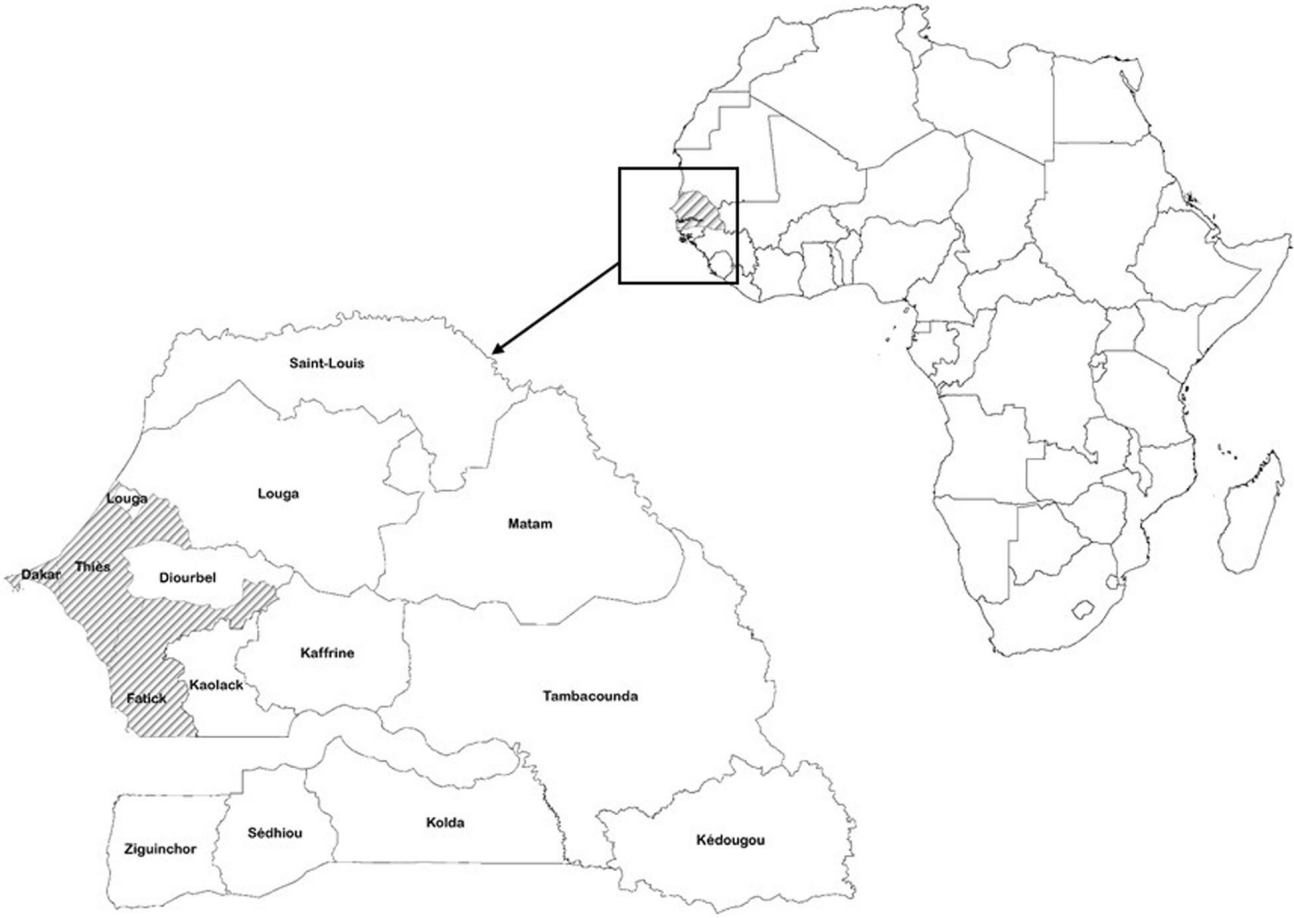
Location of Senegal and study areas. The shaded regions show the regions were the study was conducted (This map was developed by the authors using ArcMap software version 10.2.2)

This study was carried out in three adjacent regions in Senegal: Dakar, Thies and Fatick (Fig. 1). to gather data on i) consumption practices of milk and milk products, ii) knowledge and awareness of dairy farmers and milk processors of milk borne diseases and iii) practices of dairy farmers and milk processors relevant to the risk of milk contamination. Dairy farms in these three areas supply most of the milk to the population of the capital city, Dakar, which together with its metropolitan area concentrates more than 15% of the population of the country. Within these three regions, all three dairy farming systems found in Senegal- pastoral, agro-pastoral and intensive farming systems- are represented.

### Approach

There was a scarcity of data pertinent to our study question, with no previous peer-reviewed studies published, to our knowledge. For that reason, a comprehensive approach to the question that would allow us to gain insights from both quantitative and qualitative data was adopted. The data gathered through one method can be validated using the other hence strengthening the evidence generated through the study. Our strategy for data gathering combined i) a cross-sectional study of households keeping dairy cattle using a questionnaire with only closedended questions and ii) focus group discussions with dairy farmers and milk processors to collect qualitative data.

The cross-sectional survey of households keeping dairy cattle was conducted to gather data on the consumption of milk and milk products and purchasing milk products among households keeping and milking cattle in the studied areas. Specifically, questions were asked regarding: frequency of consumption of milk and different type of milk products, frequency of selling milk and milk products, boiling of milk prior to consumption and processing into milk products, frequency of processing milk into milk products, frequency of purchase of milk and milk products. The survey was carried out as part of a larger study on brucellosis described in detail elsewhere [19].

Briefly, the target population for each of the three regions was defined as ‘all bovine dairy herds present in the area’. For Dakar and Thies, recruitment was done through local veterinarians, animal health workers and community leaders. For Thies a sampling frame was obtained and 100 farms were randomly sampled. A questionnaire was designed in English, translated to French and Wolof, and administered by trained personnel using an android application: Open Data Kit (ODK). All participation was voluntary. When a household refused to participate or did not have milking cows at the time of the visit, the next household in the list was included.

Focus groups were designed and run in order to provide essential qualitative data and themes that govern consumption practices, knowledge and awareness of milk borne diseases and practices relevant to milk contamination and to help us understand the motivations and beliefs behind the practices. Focus groups discussion were conducted using a pre-designed schedule. The questions covered following domains: Milking and processing practices, perception about milk quality, knowledge and perceptions about milk borne diseases and zoonoses, the population at risk and local practices contributing to milk borne diseases. The schedule and the actual questions were tested in two pilot focus groups and revised as a result. Some questions were modified and adapted at later stages based on the discussions, in order to collect deeper information needed to answer new questions that emerged.

Two key stakeholders- milk processors and dairy farmers were selected as participants of focus groups. Community leaders in each village were approached and asked for permission prior to conducting the study. Participants were recruited through animal health workers, veterinarians and community leaders. In the study area, most of the milk processors were women and dairy farmers were men. Separate focus groups were conducted with dairy farmers and milk processors as mixing of sexes might be uncomfortable to some leading to bias. Separate focus groups were conducted among trained milk processors and untrained milk processors. A consent form was read at the beginning in the local language. It was emphasised that all participation is voluntary. Written consent was obtained. All the focus groups were conducted in local language spoken in the area which were Wolof, Pular and Serer. People with prior training and experience facilitated focus groups however; none of them had animal science background. They were fluent in the local language and lived locally ensuring the participants felt comfortable and spoke without inhibition. In addition, they were trained with a specific schedule. A translator was present who live translated the discussions to the first and second authors. All the discussions were voice recorded and coded to maintain anonymity.

### Data analysis

The data collected from the cross-sectional survey was analysed in Microsoft excel. For categorical variables, numbers and proportions of households that selected different options were calculated.

In total all the recordings were 27 h 57 min. They were transcribed and translated to English by professionals with no animal science background. The transcripts were then subjected to thematic analysis following the steps as described previously [20]. Thematic analysis is a common method used in qualitative data analysis and is flexible. It is a method used to identify and examine underlying patterns or “themes” in qualitative data. These themes are often associated with the research question. The analysis was conducted by the first author supervised by experienced researcher. The transcripts of the recordings from the study were read multiple times so that the author became familiar with it before coding. All the transcripts were imported to NVivo 12.3.0 (QSR International Pty Ltd., 2017) where they were coded. Coding involved identifying passages from each transcript associated with a specific idea and labelling them. Later the codes were developed into sub-themes followed by themes that represent whole dataset. Themes were reviewed through multiple iterations to make sure they accurately represent the data in the transcripts.

## Results

### Cross-sectional survey study

A total of 227 herds were included in the cross-sectional survey (Dakar: 60, Fatick: 111, Thies: 56 Table 1). Most of the herds kept indigenous breeds (Dakar: 60, Fatick: 111, Thies: 56). The average herd size varied from 25(2–50) in Dakar, 10 (2–25) in Fatick and 33(2–67) in Thies. The majority of the herds kept animals either in the backyard of the house or a common open area without any housing. Only 13% in Dakar, 4% in Fatick and 4% in Thies had separate housing facilities for cattle. Milk producing households in the study areas regularly consumed milk but rarely boiled it beforehand. Chilling of milk was very rare and selling raw milk was common. The most common milk product produced and consumed was curdled milk. Other milk products (butter, cheese, butter oil) were produced only by 4% of the participants. Most households that produced curdled milk do not boil milk prior to processing. It was not common to purchase dairy products among the households with dairy cattle. There was no difference between the regions for any variables studied.

**Table 1:** Socio-demographic data on the participants of focus group discussions

Category	Subcategory	Milk processors	Dairy farmers
Sex	Male	0	76
	Female	96	0
Age	18–35	32	9
	More than 35	64	67
Marital status	Married	91	75
	Widowed	7	1
Education	None	49	49
	Primary	17	22
	Secondary	8	5
	Superior	7	4
	Literacy training	10	1
	Mosque education	5	8
Dairy related activities involved	Milking	75	62
	Collecting	63	0
	Processing	96	52
	Selling	55	29

### Focus group discussions

A total of 14 focus groups were conducted with untrained milk processers, 2 with trained milk processors and 11 focus groups were conducted with dairy farmers. The demographic data on the participants are presented in Table 2.

**Table 2:** Frequency of practices related to consumption and purchase of milk and milk products from cross-sectional surveys in three dairy production areas in and around Dakar (Senegal): Dakar (*n* = 60), Fatick (*n* = 111) and Thies (*n* = 56). The number do not add up, as some questions were dependant on others

Variables	Dakar	Fatick	Thies	Total
Milk				
Frequency of consumption of milk produced by own cows (*n* = 227)				
Never	1 (1.7%)	4 (3.6%)	1 (1.8%)	6 (2.6%)
Regularly	57 (95.0%)	86 (77.5%)	51 (91.1%)	194 (85.5%)
Sometimes	2 (3.3%)	21 (18.9)	4 (7.1%)	27 (11.9%)
Frequency of chilling milk soon after collection (*n* = 227)				
Never	58 (96.7%)	103 (92.8%)	55 (98.2%)	216 (95.2%)
Sometimes	0 (0.0%)	5 (4.5%)	0 (0%)	5 (2.2%)
Regularly	2 (3.3%)	3 (2.7%)	1 (1.8%)	6 (2.6%)
Frequency of selling raw milk (*n* = 227)				
Never	0 (0%)	41 (36.9%)	1 (1.8%)	42 (18.5%)
Sometimes	5 (8.3%)	11 (10.81%)	1 (.8%)	17 (7.5%)
Regularly	55 (91.7%)	59 (53.2%)	54 (96.4%)	168 (74.0%)
Boiling milk prior to consumption (*n* = 221)				
Never	53 (89.8%)	102 (95.3%)	50 (89.2%)	205 (92.8%)
Occasionally	2 (3.4%)	2 (1.9%)	4 (7.1%)	7 (3.2%)
Sometimes	0 (0%)	0 (0%)	1 (1.8%)	1 (0.5)
Regularly	4 (6.8%)	3 (2.8%)	1 (1.8%)	8 (3.6%)
Curdled and fermented milk^a^				
Produce curdled milk at home (*n* = 227)				
Never	12 (20.0%)	19 (17.1%)	5 (8.9%)	36 (15.9%)
Occasionally	16 (26.7%)	23 (20.7%)	20 (35.7%)	59 (25.9%)
Sometimes	9 (15.0%)	23 (20.7%)	14 (25.0%)	46 (20.6%)
Regularly	23 (38.3%)	46 (41.4%)	17 (30.4%)	86 (37.9%)
Boil milk prior to producing curdled milk at home (*n* = 185)				
Never	45 (93.8%)	91 (98.9%)	49 (96.1%)	179 (96.7%)
Occasionally	2 (4.2%)	1 (1.1%)	0 (0%)	3 (1.6%)
Sometimes	0 (0%)	0 (0%)	0 (0%)	0 (0%)
Regularly	1 (2.1%)	0 (0%)	2 (3.9%)	3 (1.6%)
Frequency of consumption of curdled milk produced at home (*n* = 185)				
Never	1 (2.1%)	0 (0%)	0 (0%)	1 (0.5%)
Occasionally	15 (31.3%)	20 (21.7%)	12 (24%)	45 (24.3%)
Sometimes	11 (22.9)	22 (23.9%)	14 (28%)	45 (24.3%)
Regularly	21 (42.9%)	50 (54.3%)	24 (48%)	94 (50.8%)
Purchase commercially available curdled milk(*n* = 227)				
Never	43 (71.7%)	105 (94.6%)	43 (76.8%)	191 (84.1%)
Occasionally	17 (28.3%)	5 (4.5%)	13 (23.2%)	35 (15.4%)
Sometimes	0 (0%)	0 (0%)	0 (0%)	0 (0%)
Regularly	0 (0%)	1 (0.9%)	0 (0%)	1 (0.4%)
Produce fermented milk at home(*n* = 227)				
Never	60 (100.0%)	102 (91.9%)	56 (100.0%)	218 (96%)
Occasionally	0 (0%)	9 (8.1%)	0 (0%)	9 (4%)
Sometimes	0 (0%)	0 (0%)	0 (0%)	0 (0%)
Regularly	0 (0%)	0 (0%)	0 (0%)	0 (0%)
Purchase fermented milk from other farmers or markets (*n* = 227)				
Never	60 (100.0%)	108 (97.3%)	56 (100.0%)	258 (98.7%)
Occasionally	0 (0%)	2 (1.8%)	0 (0%)	4 (0.8%)
Sometimes	0 (0%)	1 (0.9%)	0 (0%)	1 (0.4%)
Regularly	0 (0%)	0 (0%)	0 (0%)	0 (0%)
Frequency of consumption of fermented milk (*n* = 12)				
Never	0 (0%)	0 (0%)	0 (0%)	0 (0%)
Occasionally	0 (0%)	9 (100.0%)	2 (66.7%)	11 (91.7%)
Sometimes	0 (0%)	0 (0%)	1 (33.3%)	1 (8.3%)
Regularly	0 (0%)	0 (0%)	0 (0%)	0 (0%)
Other products (butter, cheese, butter oil)				
Produce other milk products at home (*n* = 227)				
Never	60 (100.0%)	109 (98.2%)	54 (98.2%)	223 (98.2%)
Occasionally	0 (0%)	1 (0.9%)	2 (1.32%)	3 (1.3%)
Sometimes	0 (0%)	1 (0.9%)	0 (0%)	1 (0.4%)
Regularly	0 (0%)	0 (0%)	0 (0%)	0 (0%)
Boil milk prior to producing other products at home (*n* = 4)				
Never	0 (0%)	2	2	4 (100%)
Occasionally	0 (0%)	0 (0%)	0 (0%)	0 (0%)
Sometimes	0 (0%)	0 (0%)	0 (0%)	0 (0%)
Regularly	0 (0%)	0 (0%)	0 (0%)	0 (0%)
Purchase other products (*n* = 227)				
Never	60 (100.0%)	111 (100.0%)	56 (100.0%)	227 (100%)
Occasionally	0 (0%)	0 (0%)	0 (0%)	0 (0%)
Sometimes	0 (0%)	0 (0%)	0 (0%)	0 (0%)
Regularly	0 (0%)	0 (0%)	0 (0%)	0 (0%)
Frequency of consumption of other products (*n* = 4)				
Occasionally	0 (0%)	2 (100.0%)	1 (50.0%)	4 (100%)
Sometimes	0 (0%)	0 (0%)	1 (50.0%)	0 (0%)
Regularly	0 (0%)	0 (0%)	0 (0%)	0 (0%)

^a^Curdled milk is milk fermented at room temperature without adding any lactic acid bacteria and/or enzymes and fermented milk is milk fermented by adding lactic acid bacteria and/or enzymes

Themes identified in the focus group data, presented in Table 3 represent the knowledge and awareness of milk borne diseases and practices relevant to milk contamination of dairy farmers and milk processors. They give insights into what practices relevant to milk borne diseases participants follow and why.

**Table 3:** Themes and sub-themes identified after thematic analysis of the data collected through focus groups

Main themes	Sub-themes
Low awareness about milk borne diseases	Milk cannot transmit diseases
	Previous experiences and elders act as information source
	Milk consumption can cause malaria
	Lack of awareness about milk quality
	Women are more knowledgeable than men
Cultural practices and beliefs dominate decision making	Boiling causes mastitis
	Boiling affects taste and fermentation
	Trust in age old practices
	Prioritisation of need - Hierarchy and nutritional relevance dominates household rationing of milk
Positive impact of training	More awareness among trained women

### Low awareness about milk borne diseases

It was very evident that study participants trusted their animals and animal derived products. They have been keeping livestock for generations to sustain their livelihood hence they could not accept the idea that animals can carry diseases that are harmful to humans. In all the focus groups with men and women, most participants disagreed with the general concept of “milk borne diseases”. For some participants this was the first time they heard the concept. They were surprised at the question and followed up with questions to the facilitators on whether there is any harm in consuming milk.

A few of the female participants were aware that humans could get diseases through milk and meat based on their experience. However, this was not taken well by other participants and mostly older women refuted this. They were able to mention two symptoms of food poisoning: vomiting, diarrhoea, and no other food borne illnesses transmitted through animal products were mentioned in any focus group. Most participants had no knowledge about the concept of microbes except for some women and women trained in milk processing. They were aware that boiling milk kills microbes and makes milk safer for human consumption.

Participants’ lack of knowledge about the potential of milk to transmit diseases led to discussions on whether there are other ways any diseases can be transmitted to humans from animals. This topic was presented as whether humans can get sick by eating meat, managing animals, living with animals or taking care of sick animals. Most participants including men and women rejected this.

*“No. A person is not an animal. Only a person can infect his/her neighbour, not an animal”.* [Traditional milk processor, focus groups with women]

*“A person can get sick, but it is not because of animals. If someone is ill, his disease can also be found in someone who does not live with cattle”.* [Dairy farmer, focus groups with men]

The most common argument was that they have been engaged in animal husbandry for generations. Some participants mentioned the potential of beef to cause high blood pressure however, no infectious meat borne diseases were mentioned. Rabies and tuberculosis were the only diseases that male participants mentioned as zoonoses. This increased awareness about rabies is probably because of the sudden onset, severity of symptoms and clear association with dog bites. Furthermore, there have been other researchers studying rabies in the area.

There was some disparity between the knowledge of men and women regarding milk borne infections. Women seemed to know more about milk borne infections rather than other forms of zoonosis transmission. More interestingly, most of the men believed milk could not transmit any illness. Men seemed more confident in their belief of the lack of risk of acquiring the disease from milk consumption. In some focus groups with women, when the potential of milk to transmit diseases was introduced as a topic of discussion, many participants asked questions whether milk can make humans sick. Men on the other hand tend to reject the idea of zoonosis itself pointing out, in many discussions, that they never see the same symptoms in humans and animals hence no diseases can be transmitted between them.

A misconception observed in quite a few discussions concerning malaria, which was frequently mentioned when diseases transmitted through milk were discussed.

*“Yes of course, because milk can worsen malaria and fever”.* [Traditional milk processor, focus groups with women]

*“When milk is too fermented during the raining season, you can contract malaria if you consume much milk”.* [Traditional milk processor, focus groups with women]

Malaria has been an important disease in Senegal for decades. There are several organisations and government agencies trying to combat malaria. This leads to increased awareness of the disease among the public. However, people’s perception that milk and malaria are associated is interesting and the source of such a misunderstanding was not clear.

We also explored whether dairy farmers and milk processors perceive the notion of milk quality. While trained milk processors were aware about milk quality and how to conduct tests such as ‘clot on boiling test’ to examine milk quality, none of the traditional milk processors and dairy farmers had awareness about testing for milk quality. Only the obvious changes in milk due to clinical mastitis were mentioned as characteristics of ‘bad milk’. Some of the farmers mentioned that they cannot ascertain the quality of milk and it can only be tested at the processing centre where they sell it, while some farmers and processors use colour, taste and odour to assess milk quality. However all the traditional milk processors mentioned that even though they cannot say anything about the milk soon after milking, they could identify ‘bad milk’ after fermenting. Several female and male participants referred to high water content, colour changes, taste changes and poor fermentation as characteristics of poor quality milk. The only disease that affects milk quality was clinical mastitis. In addition, some superstitions were also suggested.

“*If you allow milk to rest for two days for processing and you find it compact after fermentation, it proves that it is good milk. But if you find milk that looks like water, that is bad milk”.* [Traditional milk processor, focus groups with women]

Past experiences and interaction with veterinarians and animal health workers seem to have some impact on study participants’ knowledge of food borne illnesses. Both men and women said that even though their ancestors used to eat meat from sick cows their vets told them not to do so. Similarly, previous experience of contracting diarrhoea after consuming meat from sick animals within their household or neighbourhood made them cautious.

*“Yes, we witnessed that, because after consuming this meat from sick cow, they vomited and they had diarrhoea”.* [Traditional milk processor, focus groups with women]

### Cultural practices and beliefs dominate decision-making in consumption

Milk was found to be a culturally and nutritionally important food item in the studied population. Depending upon availability, milk and milk products formed part of almost every meal. It was consumed as fresh milk without any processing in the morning and later in the day, fermented milk was consumed. Milk and fermented milk were also consumed in combination with millet for breakfast. Butter and butter oil was produced mainly during the wet season when milk is abundant and was used in cooking. A common observation from all focus groups was that cultural practice and not factual knowledge rules decision-making regarding milk processing and consumption. A common belief among traditional milk processors and dairy farmers was that boiling milk causes mastitis in cows. It was mentioned in all the focus groups.

*“In our village, it is difficult for somebody to heat the milk he has brought home. The fear is it can cause problems to the cow udders.”* [Traditional milk processor, focus groups with women]

The belief is so ingrained in the population that they are reluctant to sell the milk to any consumers whom they think might boil milk.

*“No we do not heat milk before drinking it. Even here, if a person offers you milk and you heat it, he will no more give you. It is said it causes the udder of the cow to swell*” [Dairy farmer, focus groups with men]

This was also mentioned as a challenge by trained milk processors. Trained milk processors collect milk from dairy farmers and run mini-diaries where they process milk into pasteurised milk and sweetened and/or flavoured milk. However, acquiring milk is a major challenge, as farmers are reluctant to sell milk, as they know it will be boiled.

*“It is difficult to find fresh milk because some cow or herd owners do not sell it to us as say it is not good for cows to heat milk. They say that if one heats the milk, cow will have sore breasts”.* [Trained milk processor, focus groups with women]

Another major argument identified as a reason not to boil milk prior to processing and consumption was their trust in age-old practices. Even though some of the participants are aware about the risks of milk borne pathogens they trust in what they have been doing for generations.

*“We will never get sick from drinking raw milk. That is what we have been drinking for generations”.* [Traditional milk processor, focus groups with women]

It was also mentioned that boiling milk leads to improper fermentation and does not yield good butter. Dairy farmers had the perception that boiling affects taste. Boiling was also believed to make milk unhealthy. Dairy farmers and traditional milk processors believed that milk is safest soon after milking and boiling is pointless as it is already warm. In many of the discussions, even though most are aware about the practice of heating milk they do not do it because they simply are not used to it.

*“We are not used to it [boiling milk]. Milk is already hot when you just finish milking the cow. It is hot because it directly comes from the cow.”* [Traditional milk processor, focus groups with women]

Some female participants mentioned that they boil milk prior to giving it to young children and this seems to be related to their experience when their children had diarrhoea after consuming milk. However, they would not boil milk for adult members of the household.

Milk was mentioned as a scarce commodity in some households especially during dry season. This leads to rationing in the households based on nutritional needs of the household members and social hierarchy. Women are responsible for rationing.

Cows’ milk was mentioned as a substitute for breast milk. Most women mentioned that when they wean toddlers, or when new mothers have issues producing enough breast milk, they give cows’ milk to children to maintain weight gain. Young children especially young boys are also prioritised in some households when milk is rationed.

“*Children consume it the most, also young boys because they are the ones who keep the herd. They even take milk as their breakfast in the fields after milking*”. [Traditional milk processor, focus groups with women]

Milk was also mentioned as an important part of the diet for pregnant women. Most women mentioned that eating food of nutritional value is important during pregnancy and as milk has high nutritional value they incorporate it more in their diet.

In two focus group discussions, older women mentioned that in order to ensure an easy childbirth, pregnant women near full-term are given whey.

*“If you remove the water on top of that bad milk you say is not good, and you give it to her during childbirth, she will deliver without problem”.* [Traditional milk processor, focus groups with women]

This fluid was also reported to have anthelminthic properties by participants who mentioned that they give this to children as a dewormer.

Social hierarchy was also observed when women rationed milk at home with some women saying they prioritise their father-in-law and husband before giving milk to anyone.

*“A married women I know, always gives the milk to her father-in-law first; the other family members have to wait to be served. And if the elders leave some milk, she gives it to the children”.* [Traditional milk processor, focus groups with women]

When the availability of milk is low, most women only drink milk if there is any leftover.

*“We are only serving. We only get some if it remains. Otherwise, we eat nothing”.* [Traditional milk processor, focus groups with women]

Milk was also considered important for herders. Herders sometimes consume milk fresh as soon as it is milked and give the remaining to women for household use and selling. Among the herds, which practise transhumance during the dry season, milk from their animals is crucial to satisfy their nutritional needs.

### Positive impact of training

There were several differences in how trained and untrained milk processors processed milk into milk products. The most common milk product prepared by both trained and traditional milk processors was fermented milk. Depending on the availability of milk, butter and butter oil are also produced. Some women also mentioned they received training on how to use milk to produce soap.

The traditional processing of milk to fermented milk starts with filtering milk to remove animal hair. Then the milk is poured into a vessel made from a gourd (*calabash*) or a plastic container and kept at room temperature for 2 days in summer and longer when the weather is colder. This fermented product is consumed with sugar or consumed with millet (*lakh*). Milk is never heated during this process. Most of the traditional processors reported that they never add anything such as sugar or water prior to fermenting as this will interfere with the process.

*“When you come from the pen with the milk, you filter it with the sieve and the leftover is placed in a calabash for 2 days to turn it into curdled milk”.* [Traditional milk processor, focus groups with women]

The women who received some training in milk processing and have their own mini diaries conducted processing differently. Once the milk is collected from dairy and tested for quality using ‘clot on boiling test’, it is boiled and stored in a refrigerator if they intend to sell it as milk. They also produce sweetened fermented milk. Fermentation is carried out by adding external fermenting agents, unlike the traditional method where milk is allowed to ferment naturally. Some women also used previously-fermented milk as a fermenting agent. Orange extract was added to trigger the fermentation after boiling. Sometimes the processing is carried out in refrigerated conditions unlike in the traditional process.

*“If I want to process milk into yogurt, I wear gloves, socks and a headscarf. Then I take a large pot in which I pour water. I place the small pot with the milk into the large pot and I boil it up. Later, I take it off, let it cool and then mix it. The people who taught us this method had bags with them. However, they left with them. Consequently as I do not have these bags, I just put sugar and orange blossom extract*”. [Trained milk processor, focus groups with women]

Trained women who maintain mini diaries had access to better infrastructure such as boiling equipment and refrigeration facilities. They also reported that since they started organised dairies the economic returns from selling milk and milk products have increased and there is an increased demand from customers especially during Eid. One of the hurdles they mentioned was the scarcity of milk and challenges in acquiring milk and longer distances to selling points with higher demand. Traditional milk processors focused mostly on local customers. They also mentioned milk availability during the dry season as an issue. These women also would like to establish organised diaries but lack of infrastructure, training and financial resources were mentioned as hurdles. Milk processors were more concerned about such issues and least worried about milk quality or milk borne diseases.

## Discussion

Here we examined the consumption of milk and milk products, knowledge and awareness of milk borne diseases and practices relevant to the risk of milk contamination among producers of milk and dairy products in Dakar and two nearby areas (Thies and Fatick). By understanding the actual practices and the rationale/beliefs behind them as the product of particular circumstances, this study gives us pointers on how to transform those practices. The findings from the study suggest that milk is an important part of the diet in dairy production areas around the capital. Milk is consumed mostly as raw or fermented milk, and rarely boiled. The understanding of milk borne diseases and zoonoses in general is still developing among dairy farmers and milk processors in Senegal.

A key finding from this study is the prioritisation of milk at home. This helped to identify the most vulnerable groups, which were infants, children, pregnant women and older men. Even though some women reported that they boil milk before giving it to infants and children, milk is not heat treated prior to processing to fermented milk. Fermentation is used as a preservation method and acidic pH can be detrimental to most bacteria but there are studies, which isolated *E coli, Streptococcus agalactiae, S aureus, Shigella and Salmonella spp* from fermented milk products from different places in Africa suggesting they are still at risk [21–23].

Globally there is plenty of evidence to show that consumption of unpasteurised or raw milk is the reason for milk borne illnesses and boiling is an effective way to make milk safe for human consumption [24]. However, in all the focus groups traditional beliefs were mentioned as reasons not to boil milk. When asked about the potential effects of boiling only a few, all of them trained women, mentioned reducing microbial contamination. Overall, the participants had very limited knowledge of milk borne diseases and zoonoses. In some discussions, farmers strongly refuted the idea of milk borne infections quoting a lack of the same symptoms in animals and humans suggesting they need physical verification of diseases. Such validation with symptoms in animals and humans is not always possible with milk borne diseases. Furthermore, there was some discussion around symptoms of diseases that could potentially affect milk quality suggesting limited knowledge of subclinical diseases and diseases with no obvious symptoms. Many microbiological milk borne hazards do not cause clinical disease in animals, which could be one of the reasons why they never consider milk as a potential source of illness. Some key milk borne pathogens such as *Staphylococcus aureus* and *Streptococcus agalactiae* are known to cause subclinical mastitis in bovines [25]. Lack of awareness about subclinical mastitis suggests they consume milk produced by affected cows. Another group of milk borne hazards, which can be present in milk in the absence of any symptoms in animals are chemical residues such as pesticides and antibiotics, which also were never mentioned in any discussions.

Women were more aware about milk borne illnesses and the benefits of boiling. These gender differences could be because of differences in the sharing of livestock management and household responsibilities. There are also differences in the responsibilities of men and women based on ethnic groups. However, in the study regions, women play a major role in processing milk and looking after other household members.

Despite awareness about milk borne diseases and risks of raw milk consumption, most participants did not implement any risk mitigation measures except occasionally for very young children. Their beliefs and practices passed through generations governed their decision making process. This was similar to a study conducted in Tanzania on zoonosis awareness among pastoralist communities [15]. Their faith in animal products and concepts shared by older members of the community gave them confidence about animal derived food products. Lack of knowledge about the concept of microbes might also be the reason for not implementing any risk mitigation measures. In addition, they have been practising drinking raw milk for generations and long-term exposure to small amounts of pathogens might have given the community some immunity over time, which has been proved for *Campylobacter sp* [26, 27].

This study identified some misconceptions about milk borne diseases and zoonoses in the community. Some of the misunderstandings show that the study participants receive information from unreliable sources. These beliefs are old wives tales passed on through generations. A common misconception was the association between malaria and milk consumption. Malaria is still a public health issue in Senegal and there are campaigns to increase awareness. Hence, it is likely that the community uses ‘malaria’ as a general term to denote any diseases with fever as a symptom. A similar observation was found in studies from Uganda and Mali [28, 29]. The actual infection could be brucellosis or Q-fever or other bacterial and viral diseases. This shows that there is a need to strengthen health education on zoonoses and food safety in order to make sure the public gets the right knowledge.

It was found that education and training had a huge impact on the processing of milk. Training programmes not only provided knowledge on hygienic processing and handling of milk but also an opportunity to set up their own business to improve livelihoods. Training has an effect on practices, as while there is a reluctance to boil among untrained, all of the trained processors seem to boil milk. This highlights the potential of training women to promote milk-safety practices. Trained women had a better understanding of milk borne diseases hence they handled milk hygienically. Most participants in our study were illiterate or had only basic literacy training which also may have contributed to a lack of awareness and practices without a scientific explanation.

The findings of our study are similar to the other two studies that looked at cattle farmers’ awareness about zoonosis in Senegal. A quantitative study conducted among dairy farmers found that most farmers and their household members consumed milk and milk products without any heat treatment [30]. The same study found that a significant proportion of farmers consumed meat and milk from sick cows. However, in our study only a few participants mentioned this. This difference could be because of a lack of reporting or an increase in awareness and transfer of knowledge through veterinarians and animal health workers. Similar to our study rabies was mentioned as a zoonosis.

This study was conducted with the help of translators. All the discussions were conducted in local language and the discussion was live translated to enable the first author to take notes and ask follow up questions. Even though the translator hired had no knowledge in livestock farming, he may have missed some key answers, which required deeper conversations. However, we used the transcriptions and translations for the final analysis to make sure we do not miss any discussion points. The insights we gained into knowledge, awareness and practices of key stakeholders facilitate efficient strategies for disease control. The findings suggest that in order to implement pre and post-harvest control measures for food borne illness one should use culturally adapted strategies, good communication and education.

## Conclusions

In conclusion, most people involved in dairy production and processing in the study areas of Senegal consume milk as raw and do not boil it prior to processing to milk products, the most common of which is curdled milk. The main barriers towards safe consumption and processing of milk and dairy products appear to be lack of awareness of the existence of human health hazards that may be present in cattle without causing clinical disease in animals, the reluctance of heat treatment of milk as it is often perceived as having the potential to harm the animal. There is evidence that training can improve hygienic measures practiced suggesting the need to implement training programmes for all actors involved in the milk value chain. This strategy will also help in economically empowering women, as most collectors are women. In addition, as farmers are against the concept of boiling milk the best option could be to target consumers to promote boiling prior to consumption and processing. In the case of dairy farmers and their family in order to ensure milk safety promoting hygienic practices, implementing disease control measures and innovative ways to render milk safe is needed.

## Data Availability

The datasets used and/or analysed during the current study are available from the corresponding author on reasonable request.

## Abbreviations

LMICs: Low and Middle income Countries
WHO: World Health Organisation
GDP: Gross Domestic Product
USD: United States Dollars
FAO: Food and Agriculture Organisation
ODK: Open Data Kit
